# Differences in abundance and functional intensity of characteristic microorganisms of tea plant rhizosphere soils contribute to the differentiation of tea quality in different rocky zones

**DOI:** 10.3389/fmicb.2025.1704146

**Published:** 2025-11-14

**Authors:** Xiaoli Jia, Yiling Chen, Yuhua Wang, Shaoxiong Lin, Mingzhe Li, Tingting Wang, Pengyuan Cheng, Bitong Zhu, Qi Zhang, Jianghua Ye, Haibin Wang

**Affiliations:** 1 College of Tea and Food Science/Fujian Key Laboratory of Big Data Application and Intellectualization for Tea Industry, Wuyi University, Wuyishan, China; 2 College of Life Science, Longyan University, Longyan, China; 3 College of Juncao Science and Ecology, Fujian Agriculture and Forestry University, Fuzhou, China; 4 College of Eco-Environment Engineering, Guizhou Minzu University, Guiyang, China

**Keywords:** Wuyi rock tea, rock zone differences, characteristic microorganisms, functions, tea quality

## Abstract

The formation of Wuyi rock tea’s “rock flavor” exhibits distinct zonal characteristics, potentially closely related to differences in its rhizosphere microbial communities. This study systematically analyzed rhizosphere soil and leaf samples from tea plants in authentic rocky zone (ZY), semi-rock zone (BY), and continent zone (ZC) zones to uncover the microbiological mechanisms influencing tea quality. Results revealed significant gradient differences in tea quality indices (catechin, theanine, and caffeine content) following ZY > BY > ZC. Soil physicochemical analysis revealed that ZY exhibited the highest contents of available nitrogen and phosphorus, while ZC demonstrated superior organic matter content. Microbial community analysis indicated that ZY possessed the highest microbial functional diversity but the lowest network complexity, with community construction dominated by random processes. Through machine deep learning, the study identified *Obscuribacteraceae* and *Psoroglaena* as two key characteristic microbial genera, whose abundance showed significant positive correlations with tea quality indices. Functional prediction analysis further indicated that these two genera were significantly enriched in specialized pathways such as photosynthesis and lichenization. Moreover, the abundance of these characteristic microorganisms showed significant positive correlations with their corresponding functional intensities, soil physicochemical indices, and tea quality indices. This study elucidated the soil microbial ecological basis for the formation of Wuyi rock tea quality across different rock zones from the perspectives of microbial community construction mechanisms and functional property. It provides theoretical support for understanding tea plant–soil-microorganism interactions and precision management in tea plantations.

## 1 Introduction

As a globally recognized member of the three major non-alcoholic beverage categories, the mechanisms governing tea quality formation remain a central focus in tea science. Wuyi rock tea celebrated for its distinctive “Yan yun” (rocky flavor), exhibits quality traits inherently linked to its unique geographical environment (Wu et al., 2024). Traditionally, its production zones have been classified into authentic rocky zone (ZY), semi-rock zone (BY), and continent zone (ZC) based on geological conditions, and there are significant differences in the organoleptic qualities and market prices of the tea from these three zones (Xiao, 2017). However, the microbial ecological mechanisms underlying the formation of such differences have not been systematically elucidated. With the development of omics technology, it is possible to analyze the formation mechanism of tea quality differences from the perspective of microbial communities, which is not only of great theoretical value, but also important in the practice of tea production.

Wuyi rock tea are mainly manifested in the unique “rocky flavor,” which is the result of the common action of different types of secondary metabolites in tea leaves (Chen et al., 2018). It has been shown that tea polyphenol and theanine contents of tea leaves in ZY are significantly higher than those of other zones, which is closely associated with its special ecological environment (Ye et al., 2024). Zhou et al. (2019) found that the soil in ZY has a unique mineral composition, especially higher potassium and magnesium contents, which may affect the metabolic pathways of the tea plant. However, it is difficult to fully explain the zone variation in rock tea quality by soil physicochemical properties alone, because neighboring tea plantations may still have significant differences in tea quality despite similar soil types (Wang et al., 2024). In recent years, research on plant–soil-microorganism interactions has provided new perspectives for understanding tea quality differences. Rhizosphere microorganisms can affect plant growth and metabolism in a variety of ways, including promoting nutrient uptake, regulating hormone balance, and inducing systemic resistance (Saeed et al., 2021). In tea plant rhizosphere soil ecosystems, specific microbial taxa are associated with the accumulation of tea quality components (Jibola-Shittu et al., 2024). For instance, Zhou et al. (2022) revealed that certain *Bacillus* bacterial genus can influence theanine synthesis and affect tea quality by regulating nitrogen metabolism. Shao et al. (2024) found that tea plants planted in large areas on flat land were susceptible to the aggregation of a large amount of *Trichoderma, Penicillium, Talaromyces* of pathogenic fungi in rhizosphere soil, reducing the health of tea plants and affecting their growth. Huang et al. (2022) found that *Bacillus amyloliquefaciens* BM1 was screened from rhizosphere soil of tea plants, then inoculated into tea plant roots, which could effectively promote the growth of tea plants and improve tea quality. It is evident that there is a close relationship between tea quality and soil microorganisms, especially some characteristic microorganisms. However, whether the formation of quality differences in tea leaves across distinct rock zones is associated with variations in soil microorganisms remains unreported.

It is hypothesized that distinct microbial taxa in ZY, BY, and ZC zones are differentially associated with tea quality indices, and that deterministic processes dominate community assembly in high-quality zones. Accordingly, tea plants from ZY, BY and ZY tea plantations in Wuyi Mountain were used as experiment materials. Rhizosphere soil and leaves of tea plants from 30 tea plantations in different rocky zones (10 tea plantations in each rock zone), which were used to systematically investigate the differences in tea quality in different rocky zones, soil physicochemical properties and microbial community structure, through the integration of tea quality indices, the rhizosphere soil physicochemical analysis, high-throughput sequencing, and the bioinformatic analysis. In this study, characteristic microorganisms of rhizosphere soils of tea plants in different rocky zone were screened, and the microbial community characteristics were correlated with soil physicochemical properties and tea quality components through functional prediction, which brought a new insight to understand the microbial mechanism of the formation of “rocky flavor.” This study helps to deepen the understanding of tea-soil-microbial interactions and provides a important basis for the scientific management of tea plantations.

## 2 Materials and methods

### 2.1 Experimental materials

Wuyishan City (longitude 117°37′22 ~ 118°19′44, latitude 27°27′31″ ~ 28°04′49″) is a major tea-producing area in Fujian Province, China, located in the northwestern part of the Province, with a total area of about 2,813 square kilometers in the city. Wuyishan City has an average elevation of 650 meters, with acidic red soil as the predominant soil type. Annual precipitation averages approximately 1,900 millimeters, and the average temperature is about 18 °C. According to the traditional division and the “Geographical Indication Product Wuyi Rock Tea” (GB/T 18745-2006), Wuyi Rock Tea production zone is divided into three categories according to soil, geomorphology and microclimate, authentic rocky zone (ZY), semi-rock zone (BY), and continent zone (ZC).

**Figure 1:**
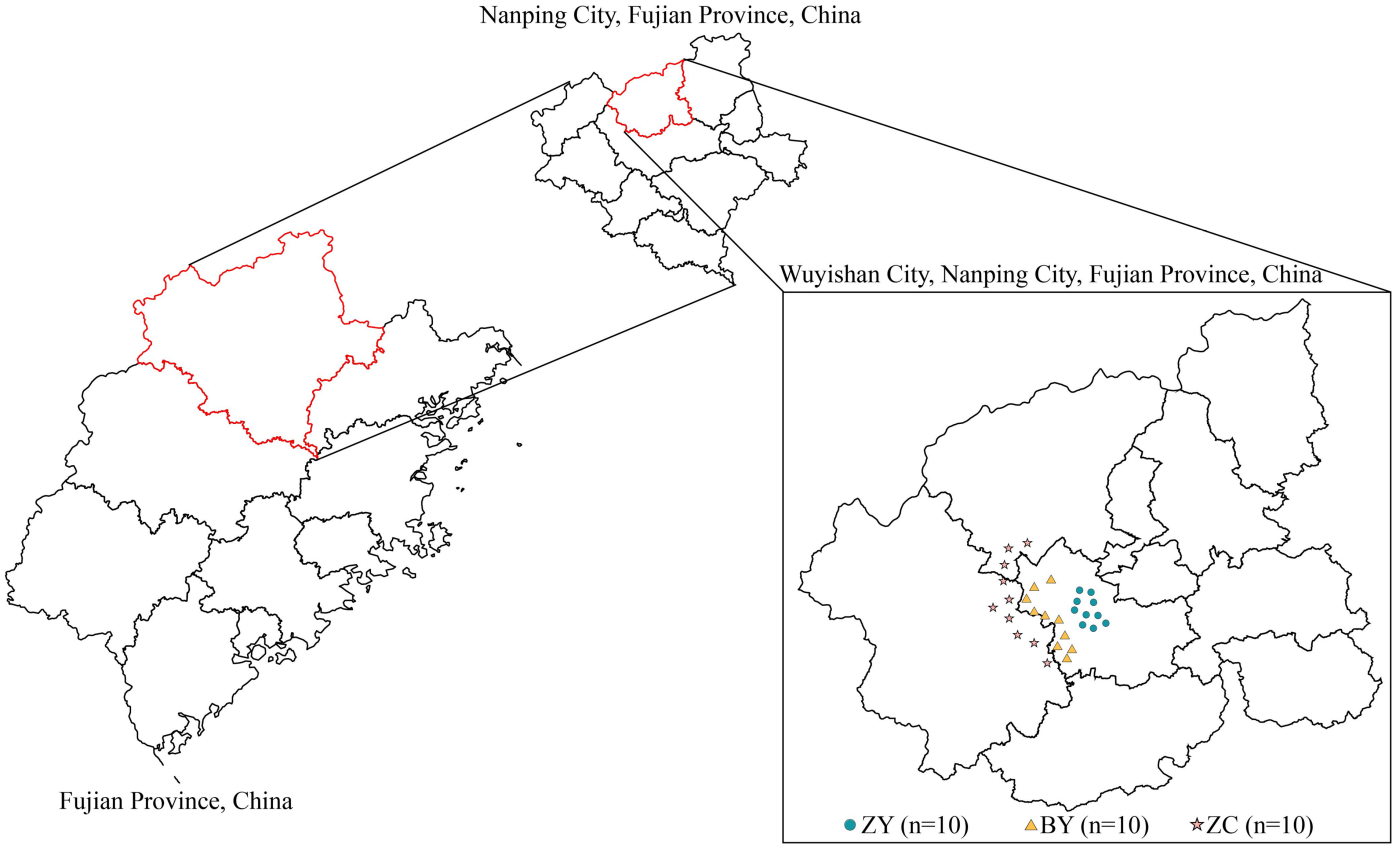
Distribution map of sampling points of 10 authentic rocky zones (ZY), 10 semi-rock zones (BY) and 10 continent zones (ZC) tea plantations in Wuyishan City, Fujian Province, China.

In this study, leaves and rhizosphere soils of tea plants were collected from 30 tea plantations (10 each from ZY, BY and ZC) (Figure 1). The selected tea plant varieties are all Wuyi Rougui (*Camellia sinensis*). Among them, part of the rhizosphere soil was utilized for measuring basic soil physicochemical indices, part of it was used for high-throughput sequencing to analyse soil bacteria and fungi, while tea plant leaves were utilized for measuring tea quality indices. Rhizosphere soil sampling was performed using the S-shaped sampling method, which consisted of randomly selecting eight tea plants, removing the dead leaves on the soil surface, gently digging out the tea plant root, shaking off the soil adhering to the surface of the root system, and then collecting the soil that was still adhering to the root system, which was the rhizosphere soil sample of the tea plants, and then mixing them together sufficiently to form a replicate (Feng et al., 2024). One bud and two leaves of tea plant at the same time were collected, and one replicate was made after intensive mixing (Huo et al., 2021). Three independent replicates were collected from each tea plantation, with each replicate spaced at least 50 meters apart. The results were averaged.

### 2.2 Determination of tea plant leaf quality indices

The study of Tang et al. (2023) was referred to measure quality indices, including tea polyphenols, caffeine and theanine of tea plant leaves from tea plantations in 30 different rocky zones. Briefly, tea plant leaves were killed at 105 °C for 15 min, dried at 80 °C until a constant weight, ground into fine powder and sieved through a 60-mesh sieve before quality indice measurement. Tea polyphenols were measured through the folin–ciocalteu colorimetric method, 5 mL of methanol solution was added to 1 g of the sample, heated at 70 °C for 10 min in a water bath, centrifuged, and 1 mL of the supernatant was collected, and 5 mL of folin–ciocalteu reagent was added to the sample. Next, 4 mL of 7.5% sodium carbonate (Na₂CO₃) solution was added to the sample, followed by a 60 min standing period. The absorbance at 765 nm was measured and quantified against a gallic acid standard curve. High performance liquid chromatography (HPLC) was used to quantify theanine, with sample pretreatment involving the addition of 100 mL boiling distilled water to 1 g of the sample. After a 100 °C water bath for 30 min, the mixture was filtered, fixed, passed through a 0.45 μm filter membrane and then used for HPLC determination and quantified by the theanine standard curve. Caffeine was determined by colorimetric method, 250 mL of boiling distilled water was added to 2 g of the sample in a 100 °C water bath for 45 min. The mixture was filtered and fixed, i.e., 5 mL of the filtrate was added 2 mL of hydrochloric acid (0.01 mol/L), fixing to 50 mL, standing, and filtration. Quantification was performed by measuring absorbance at 274 nm and referencing a caffeine standard curve; all indices were determined with three independent replicates per sample.

### 2.3 Determination of basic physicochemical indices of soil

The physicochemical indices of tea plant rhizosphere soil in different rocky zones were mainly determined as pH, organic matter content, total nitrogen, total potassium, total phosphorus, available nitrogen, available potassium and available phosphorus content. Three replicates were used for each sample, and the specific measurement methods were referred to Marion et al. (2022). Briefly, pH was measured potentiometrically at water-to-soil ratio of 2.5:1. Organic matter content was measured through high temperature oxidation of potassium dichromate and concentrated sulphuric acid to the soil, then titrated using ferrous sulphate solution. The total nitrogen content was measured through high temperature digestion of the soil with concentrated sulfuric acid followed by Kjeldahl nitrogen determination. Total phosphorus content was measured through molybdenum-antimony anti-spectrophotometry after NaOH fusion, and total potassium through flame photometry. Available nitrogen was measured through NaOH leaching-HCl titration, available phosphorus through NaHCO₃ extraction followed by molybdenum-antimony anti-spectrophotometry, and available potassium through ammonium acetate extraction and flame photometry.

### 2.4 High-throughput sequencing and bioinformatics analysis of soil microorganisms in the rhizosphere of tea plant

#### 2.4.1 High-throughput sequencing of rhizosphere soil microorganisms of tea plant

Soil microbial DNA was extracted with the Bio-Fast Soil Genomic DNA Kit (BioFlux, Hangzhou, China), purified using TianGen’s Gel Recovery Kit, quantified by UV spectrophotometry, and subsequently used for PCR amplification. Soil bacterial 16S rDNA was amplified according to the study of Hussain et al. (2021). The primers used for PCR amplification were 338\u00B0F (ACTCCTACGGGGAGGCAGCAG) and 806R (GGACTACHVGGGTWTCTAAT). The 25 μL PCR system contained 12.5 μL 2 × Taq Plus Master Mix, 3 μL BSA, 1 μL forward primer, 1 μL reverse primer, 2 μL DNA (30 ng), and 5.5 μL ddH₂O. Thermal cycling conditions were: pre-denaturation at 94 °C for 5 min; 30 cycles of 94 °C/30 s, 50 °C/30 s, and 72 °C/60 s; and final extension at 72 °C for 7 min. Fungal ITS rDNA amplification followed the protocol of Tajiki et al. (2021). PCR primers of soil fungi were ITS1 (CTTGGTCATTTAGAGGAAGTAA) and ITS2 (TGCGTTCTTCATCGATGC). The PCR system contained 2 μL DNA (30 ng), 1 μL forward primer, 1 μL reverse primer, 3 μL BSA, 12.5 μL 2xTaq Plus Master Mix, 5.5 μL dd H_2_O. The PCR program included pre-denaturation for 5 min at 95 °C, denaturation for 45 s at 95 °C, annealing for 50 s at 55 °C, and extension for 45 s at 72 °C for 34 cycles, finally maintaining 10 min at 72 °C. Bacterial and fungal PCR products were purified with Agencourt AMPure XP (Beckman Coulter, Inc., United States), library construction using the NEB Next Ultra II Kit (New England Biolabs, Inc., United States), and sequenced at Beijing Allwegene Technology Co. Ltd. (Beijing, China) on Illumina Miseq/Nextseq 2000/Novaseq 6,000 platforms (Illumina, Inc., United States) with PE250/PE300 paired-end sequencing.

#### 2.4.2 Bioinformatics analysis

Sequenced bacterial and fungal gene sequences were analysed via Illumina Analysis Pipeline (v2.6). Raw Fastq data were first filtered with Trimmomatic software (v0.36) to remove ambiguous bases (N) and low-quality reads (Q < 20), then spliced using Pear (v0.9.6; min overlap = 10 bp, *p* = 0.0001) (Zhang et al., 2014). After removing sequences < 120 bp with Vsearch software (v2.7.1), chimeras were eliminated via UCHIME against the UNITE database (v8.2) to generate clean tags (Rognes et al., 2016). OTU were clustered at 97% similarity using UPARSE in Vsearch software (v2.7.1) (Edgar, 2013), and taxonomically annotated via BLAST against UNITE (v8.2; e-value = 1e-5) (Abarenkov et al., 2010; Ye et al., 2006). Bacterial and fungal functions were predicted using FAPROTAX and FUNGuild, respectively.

Ecological diversity analyses were conducted using OTUs and their abundance data. Rarefaction curve (sequences vs. observed OTUs) were generated via sequential random sampling to evaluate sequencing depth. Shannon-Wiener curves were plotted to reflect diversity saturation across sequencing depths for each sample. Rank-abundance curves (OTU rank vs. sequence count) illustrated community evenness, while species accumulation curves (sample size vs. new OTU emergence rate) assessed sampling completeness. *α*-Diversity indices (Shannon, Simpson, Chao1, PD whole tree) were computed in QIIME1 (v1.8.0), and visualizations were generated using Rstudio (R version 4.2.3).

### 2.5 Statistical analysis

Raw data were initially organized in Microsoft Excel 2021, then processed and statistically analysed using IBM SPSS Statistics software (v26) and Rstudio (v4.2.3). were used to process and statistically analyse the data. All indices (content or abundance) were presented as mean ± standard deviation (means±SD). Rstudio software (v 4.4.3) was used for data analysis and graphical production. The ggplot2 3.5.1, vegan 2.6.10 and ggpubr 0.6.0 were used for α-diversity analysis, and tidyverse 2.0.0 and ellipse 0.5.0 for *β*-diversity analysis. Symbiotic network analysis (dplyr 1.1.4 and Hmisc 5.2.2) and neutral community models (minpack. lm 1.2.4, stats4 4.4.2, and grid 4.4.2) were used to evaluate the differences in microbial diversity in tea rhizosphere soils in different rock zones. Kruskal Wallis test (*p* < 0.05) and linear discriminant analysis effect size (LEfSe, microeco 1.4.0 and stringr 1.5.1) were used to screen for bacteria or fungi with significant differences in abundance in the rhizosphere soil of tea plants in different rock zones. Heatmap (pheatmap 1.0.12) was used to analyze the abundance differences of bacteria or fungi in different samples. Orthogonal Partial Least Squares Discriminant Analysis (OPLS-DA) model and Random Forest Machine Deep Learning were used to screen and distinguish characteristic bacteria or fungi in the rhizosphere soil of tea plants in different rock zones. Among these, the R packages used for OPLS-DA model construction were ropls and mixOmics. The R package used for random forest machine learning was tidymodels 1.1.1 (The dataset was split into a training set and an independent test set at a 7:3 ratio. Standardized preprocessing was performed using tidymodels’ recipe, with 5-fold cross-validation applied. Bacterial tuning parameters included mtry: 3–4, trees: 700–900, min_n: 2–3. Fungal tuning parameters included mtry: 4, trees: 200–250, min_n: 1–2.). The R packages used for SHAP swarm map production were Shapviz 0.9.3, fastshap 0.1.1, and iml0.11.1. The functional prediction and enrichment were used to analyze functional and intensity differences of characteristic bacteria or fungi in tea plant rhizosphere soil in different rock zones. Among these, the R package used for KEGG pathway enrichment analysis was clusterProfiler 4.8.2. The R package used for Sankey diagram production was ggalluvial 0.12.5. The R package used for box plot production was tidyr 1.3.1.

## 3 Results

### 3.1 Analysis of leaf quality indices of tea plant in different rocky zones

**Figure 2:**
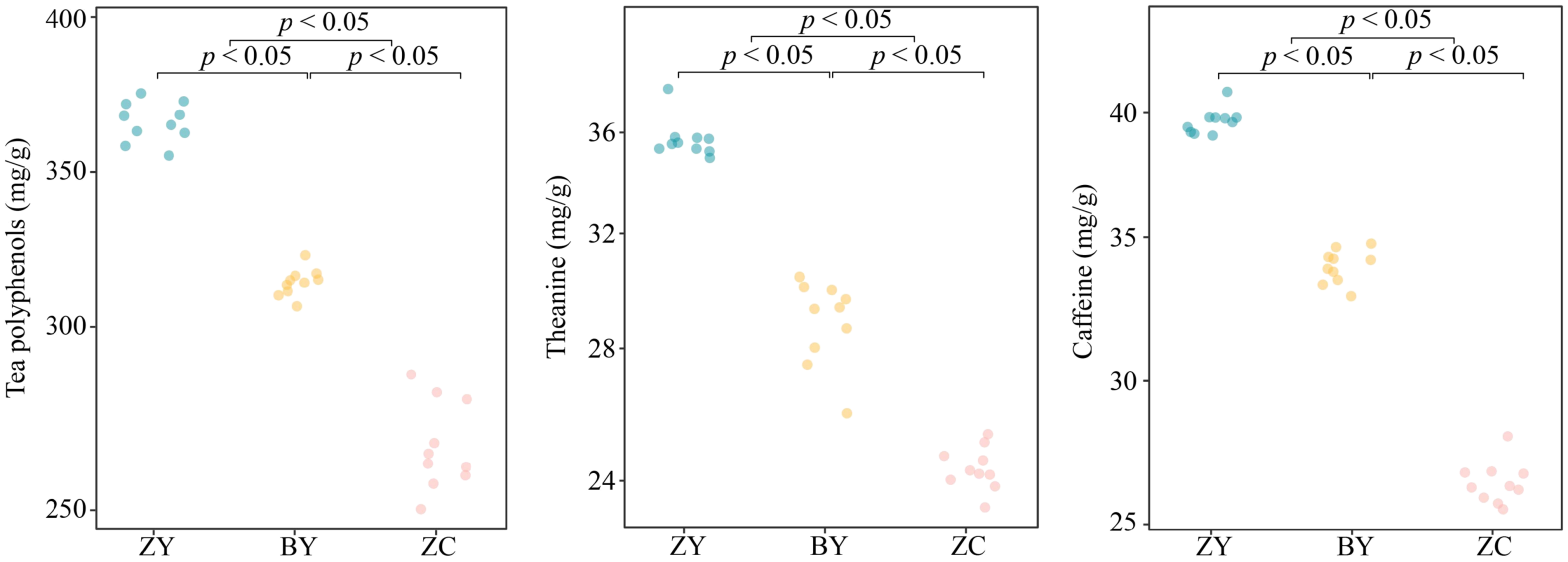
Analysis of leaf quality indices (tea polyphenols, theanine, and caffeine contents) in authentic rocky zone (ZY), semi-rock zone (BY) and continent zone (ZC) tea plantations.

In this study, quality indices of tea plant leaf were measured for each of 10 tea plantations from authentic rocky zone (ZY), semi-rock zone (BY) and continent zone (ZC). The results indicated (Figure 2) that the tea polyphenol, theanine and caffeine contents ranged from 355.77, 34.93, 39.03 to 378.40, 37.87, 40.90 mg/g in ZY tea plant leaves, respectively, and from 306.13, 25.97, 32.83, to 322.10, 30.43, 34.77 mg/g in BY tea plant leaves, respectively, while from 250.27, 23.27, 26.13 to 286.13, 25.33, 28.27 mg/g in ZC tea plant leaves, respectively. Further analysis revealed that in the leaf quality indices, ZY tea plants were significantly greater than BY and ZC, while BY was significantly greater than ZC. It is evident that there were significant differences in the quality indices of tea leaves from different rocky zones, with ZY having the highest content of quality indices, followed by BY, and ZC having the smallest.

### 3.2 Analysis of basic physicochemical indices of tea plant rhizosphere soils in different rocky zones

**Figure 3:**
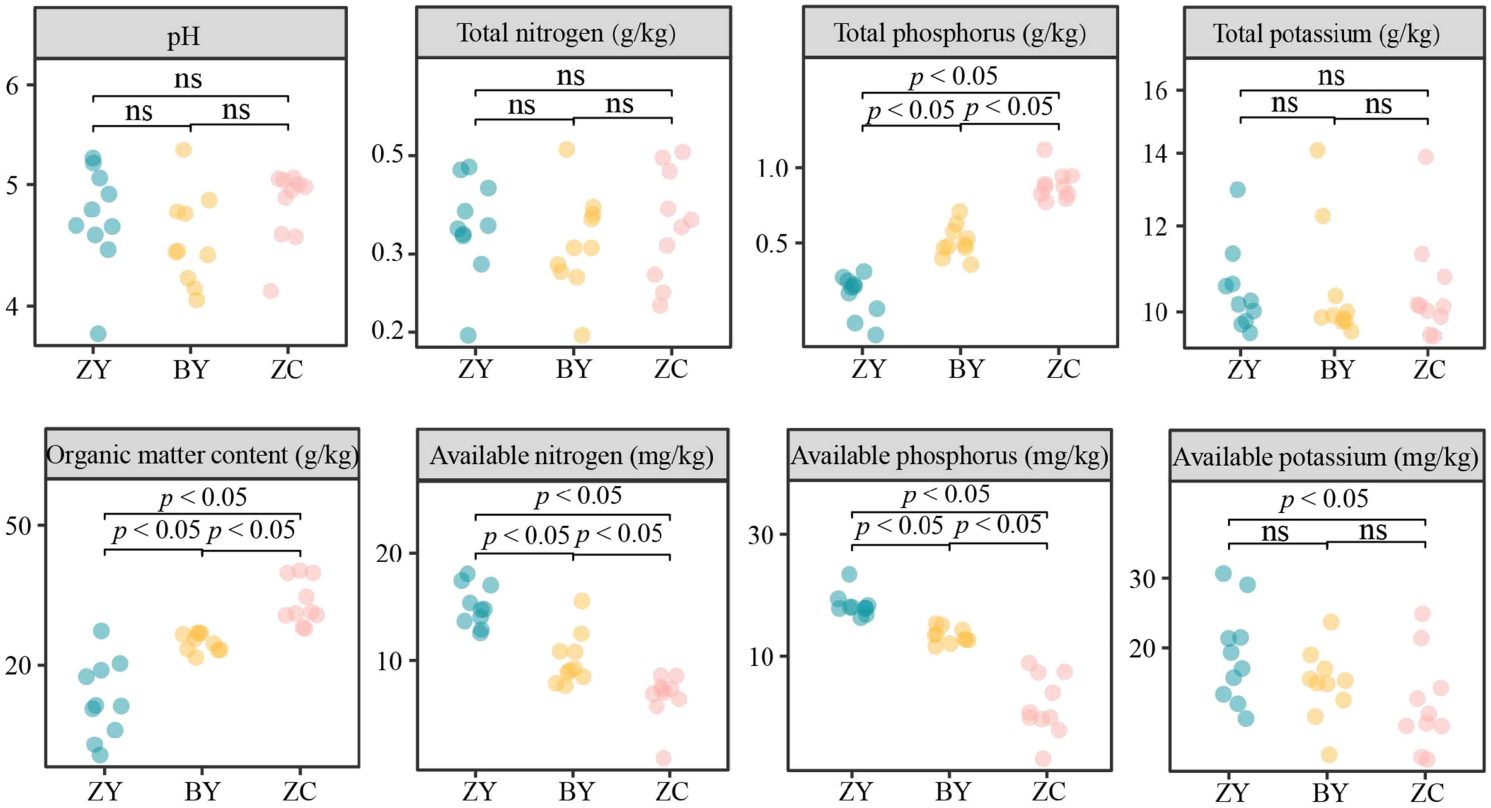
Analysis of basic physicochemical indices of tea rhizosphere soil in authentic rocky zone (ZY), semi-rock zone (BY) and continent zone (ZC) tea plantations – pH, total nitrogen, total phosphorus, total potassium, organic matter, available nitrogen, available phosphorus, and available potassium contents.

The results of the basic physicochemical indices of tea plant rhizosphere soils in different rocky zones indicated that (Figure 3), the pH value and the contents of soil total nitrogen and total potassium of tea plantations in different rocky zones did not differ significantly (*p* > 0.05), whereas the contents of total phosphorus, organic matter, available nitrogen, available potassium, and available phosphorus were significantly different (*p* < 0.05). Among them, the contents of total phosphorus and organic matter presented ZC > BY > ZY, available nitrogen and available phosphorus presented ZY > BY > ZC, while the differences of available potassium contents between ZY and BY, between ZC and BY were not significant, but ZY was significantly larger than ZC. It is evident that there were significant differences between the basic physicochemical indices of rhizosphere soils of tea plants in different rocky zones, especially in total phosphorus, organic matter, available nitrogen, available potassium, and available phosphorus.

### 3.3 Basic information on rhizosphere soil microbial communities of tea plant in different rocky zones

Soil bacteria and fungi of tea plant in different rocky zones were determined. A total of 1,563,559 Mb of raw tags were obtained after sequencing of bacteria, and a total of 1,479,754 Mb of clean tags were obtained after data cleaning (Supplementary Table S1), and the sequence lengths ranged from 200 to 540 bp, especially from 380 to 440 bp (Supplementary Table S2). After cluster analysis of clean tags, a total of 56,609 OTUs were gained, among which, the OTU number of tea plant rhizosphere soil bacteria from different rocky zones was distributed between 698 and 2,542, and there were 90 similar OTUs (Supplementary Table S3). A total of 2,177,411 Mb of raw tags were obtained after fungal sequencing, and 2,078,142 Mb of clean tags were obtained after data cleaning (Supplementary Table S4), and the sequence lengths were mainly ranged from 0 to 540 bp, especially from 200 to 320 bp (Supplementary Table S5). After cluster analysis of clean tags, 22,590 OTUs were gained. Among them, the OTU number of tea plant rhizosphere soil fungi in different rocky zones was distributed between 345 and 995, and there were 33 similar OTUs (Supplementary Table S6).

The rarefaction (Supplementary Figure S1A), Shannon-Wiener (Supplementary Figure S1B), rank-abundance (Supplementary Figure S1C), and species accumulation curves (Supplementary Figure S1D) for bacterial OTUs all plateaued, demonstrating that sequencing depth and sample size were sufficient to represent the tea plant rhizosphere bacterial community. Further sampling would result in negligible new species discovery. Secondly, the Simpson (0.87 ~ 1.00, mean 0.98) and Shannon (5.50 ~ 9.51, mean 8.49) indices of the OTUs indicated that soil bacterial community was relatively rich in diversity, while the Chao1 (distribution range 1119.67 ~ 3473.80, mean 2625.09) and PD whole_tree (distribution range 73.86 to 151.22, mean 120.91) indices illustrated that significant difference existed in the abundance of bacterial community among the samples (Supplementary Table S7).

Fungal community sequencing data were assessed via rarefaction (Supplementary Figure S1E), Shannon-Wiener (Supplementary Figure S1F), rank-abundance (Supplementary Figure S1G), and species accumulation curves (Supplementary Figure S1H). All curves reached saturation as sequence counts or sample size increased, confirming that sequencing depth and sample size were sufficient to capture the tea plant rhizosphere fungal community, with minimal new fungal species expected from further sampling. The Simpson (0.84 ~ 0.97, mean 0.92) and Shannon (3.67 ~ 6.90, mean 5.54) indices indicated that high diversity within the fungal community, while the Chao1 (699.58 ~ 1462.5, mean 5.54) and PD whole_tree (82.23 to 194.82, mean 153.10) indices revealed significant variations in the abundance of fungal communities among the samples (Supplementary Table S8).

### 3.4 Analysis of the diversity of rhizosphere soil microbial communities of tea plant in different zones

**Figure 4:**
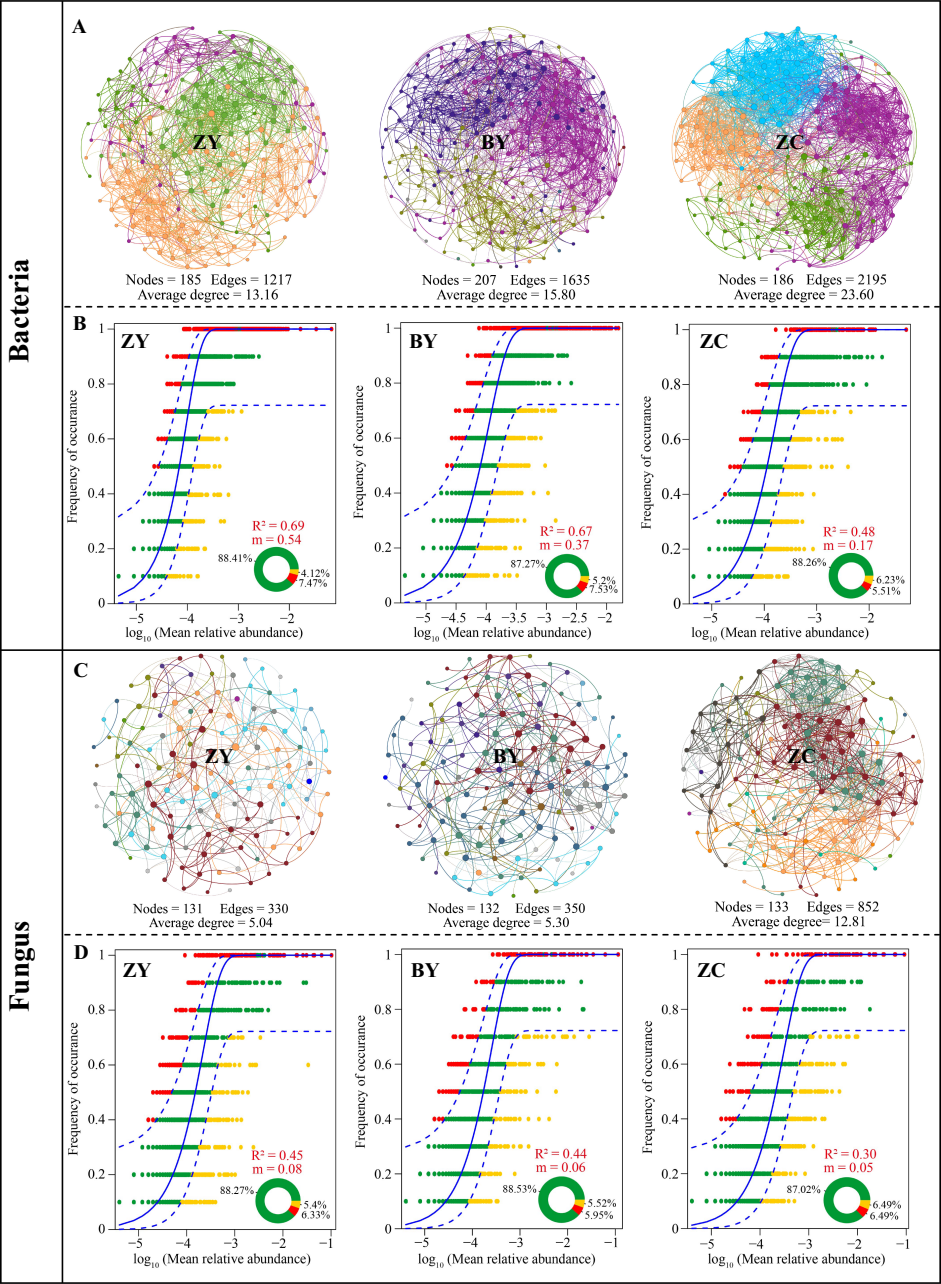
Symbiotic network analysis and neutral community models to evaluate the differences in microbial diversity in tea plant rhizosphere soil in authentic rocky zone (ZY), semi-rock zone (BY) and continent zone (ZC) tea plantations. **(A)** Symbiotic network diagram of the bacterial community; **(B)** Neutral community model of the bacterial community; **(C)** Symbiotic network diagram of the fungal community; **(D)** Neutral community model of the fungal community.

On the basis of the aforementioned analysis, this study analysed the complexity and stability of rhizosphere soil microbial communities of tea plant in different rocky zones using symbiotic network and neutral community models. Among them, the symbiotic network analysis of bacterial communities indicated (Figure 4A) that the average degree of symbiotic network nodes of tea plant rhizosphere soil bacterial communities in different rocky zones presented ZC (23.60) > BY (15.80) > ZY (13.16). It is evident that the diversity of tea plant rhizosphere soil bacterial communities in ZC was the most complex, while ZY was relatively simpler. The neutral community model analysis of tea plant rhizosphere soil bacteria in different rocky zones revealed (Figure 4B) that the *R*^2^ and migration rate (m) of ZY were the largest, 0.69 and 0.54, respectively; BY was the second largest, 0.67 and 0.37, respectively; and ZC was the smallest, 0.48 and 0.17, respectively. Soil bacterial community of tea plant in ZY was the most affected by stochastic process, and the bacterial community was most easy to spread and the lowest stability, while soil bacterial community in ZC was not easy to spread and more stable.

The symbiotic network analysis of fungal communities indicated (Figure 4C) that the average degree of symbiotic network nodes of soil fungal communities of tea plant in different rocky zones still presented ZC (12.81) > BY (5.30) > ZY (5.04). It is evident that from the perspective of fungal communities, the diversity of soil fungal communities of tea plant in ZC was still the most complex, while ZY was simpler. The neutral community model analysis of soil fungi in different rocky zones also revealed (Figure 4D) that the *R*^2^ and migration rate (m) of ZY were the largest, 0.45 and 0.08, respectively; BY was the second largest, 0.44 and 0.06, respectively; and ZC was the smallest, 0.30 and 0.05, respectively. It is evident that soil fungal community in ZY was still most influenced by the stochastic process and was the most likely to spread and the least stable, while soil fungal community in ZC was less likely to spread and more stable.

### 3.5 Screening of rhizosphere soil differential microorganisms of tea plant in different rocky zones and analysis of their abundance changes

**Figure 5:**
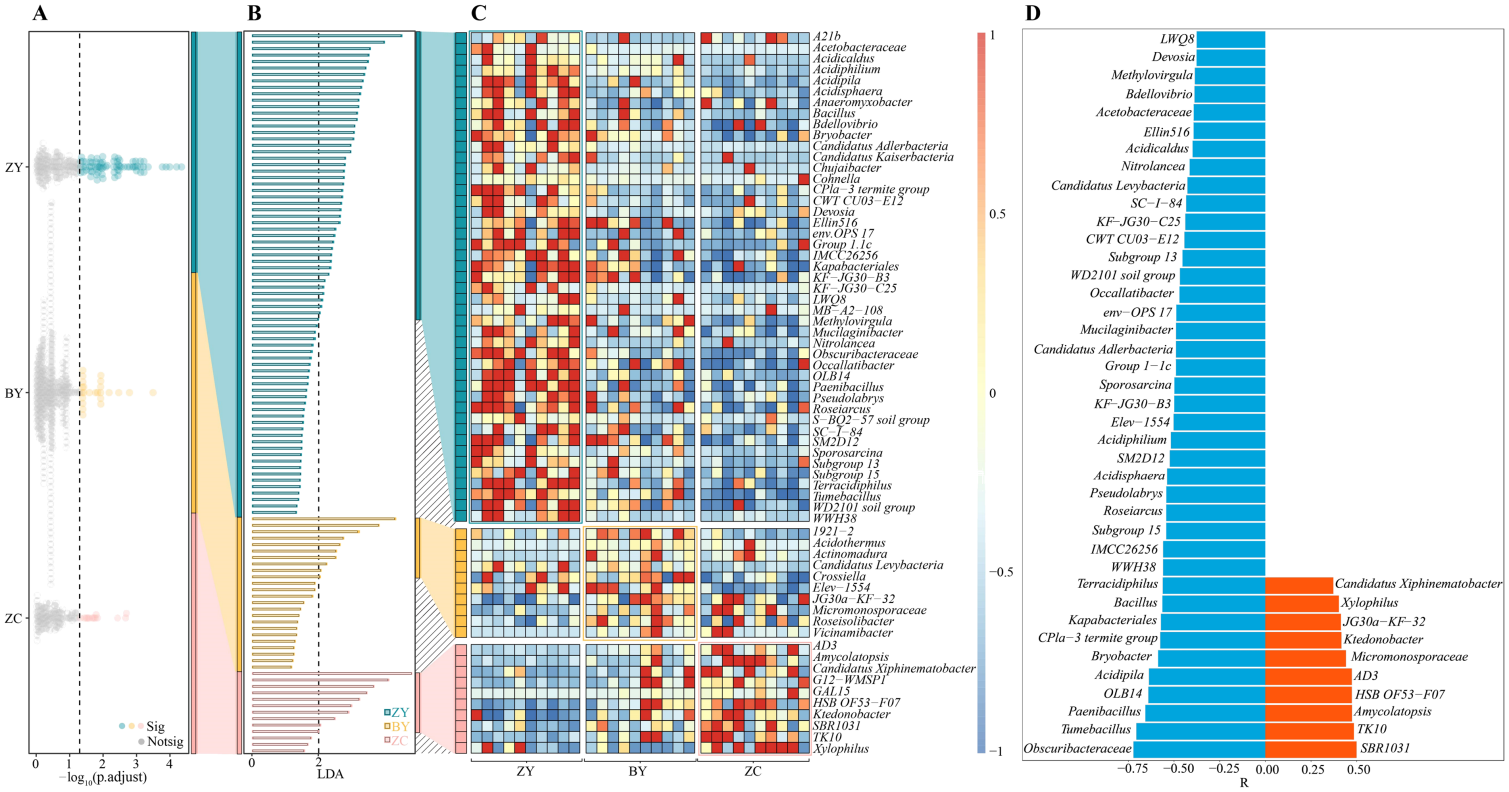
Kruskal Wallis test and linear discriminant analysis effect size (LEfSe) to screen for bacteria with significant differences in abundance in tea plant rhizosphere soil in authentic rocky zone (ZY), semi-rock zone (BY) and continent zone (ZC) tea plantations. **(A)** Kruskal-Wallis test for average bacterial abundance; **(B)** LEfSe analysis of bacteria that significantly distinguished ZY, BY, and ZC; **(C)** Heat map analysis of changes in differential bacterial abundance; **(D)** Trend analysis of changes in differential bacterial abundance (a negative value of relative coefficient (R) indicates a significant decreasing trend in bacterial abundance from the authentic zone to the semi-rock zone to the continent zone, whereas a positive value indicates a significant increasing trend).

The above analysis found that significant differences existed in the microbial community structure and diversity in the rhizosphere soils of tea plants in different rocky zones. Accordingly, in-depth study screened the microorganisms that differed significantly in the rhizosphere soils of tea plants in different rocky zones. First, the Kruskal-Wallis test was utilized to analyse soil bacterial community in different rocky zones in this study, and the results indicated (Figure 5A) that 112 bacterial genera were significantly different in abundance. Analysis using LEfSe’s LDA method (*p* < 0.05 and LDA > 2) revealed that 65 bacterial genera out of 112 genera could be significantly differentiated between ZY, BY and ZC (Figure 5B), and they were obviously different in abundance (Figure 5C). Further trend analysis of the 65 bacterial genera revealed (Figure 5D) that a total of 50 bacterial genera indicated regular changes from the authentic rocky zone to the semi-rock zone to the continent zone, of which 10 bacterial genera indicated a significant upward trend and 40 bacterial genera indicated a significant downward trend. It is evident that the differences in rock zones resulted in significant changes in bacterial abundance in the rhizosphere soil of tea plants.

**Figure 6:**
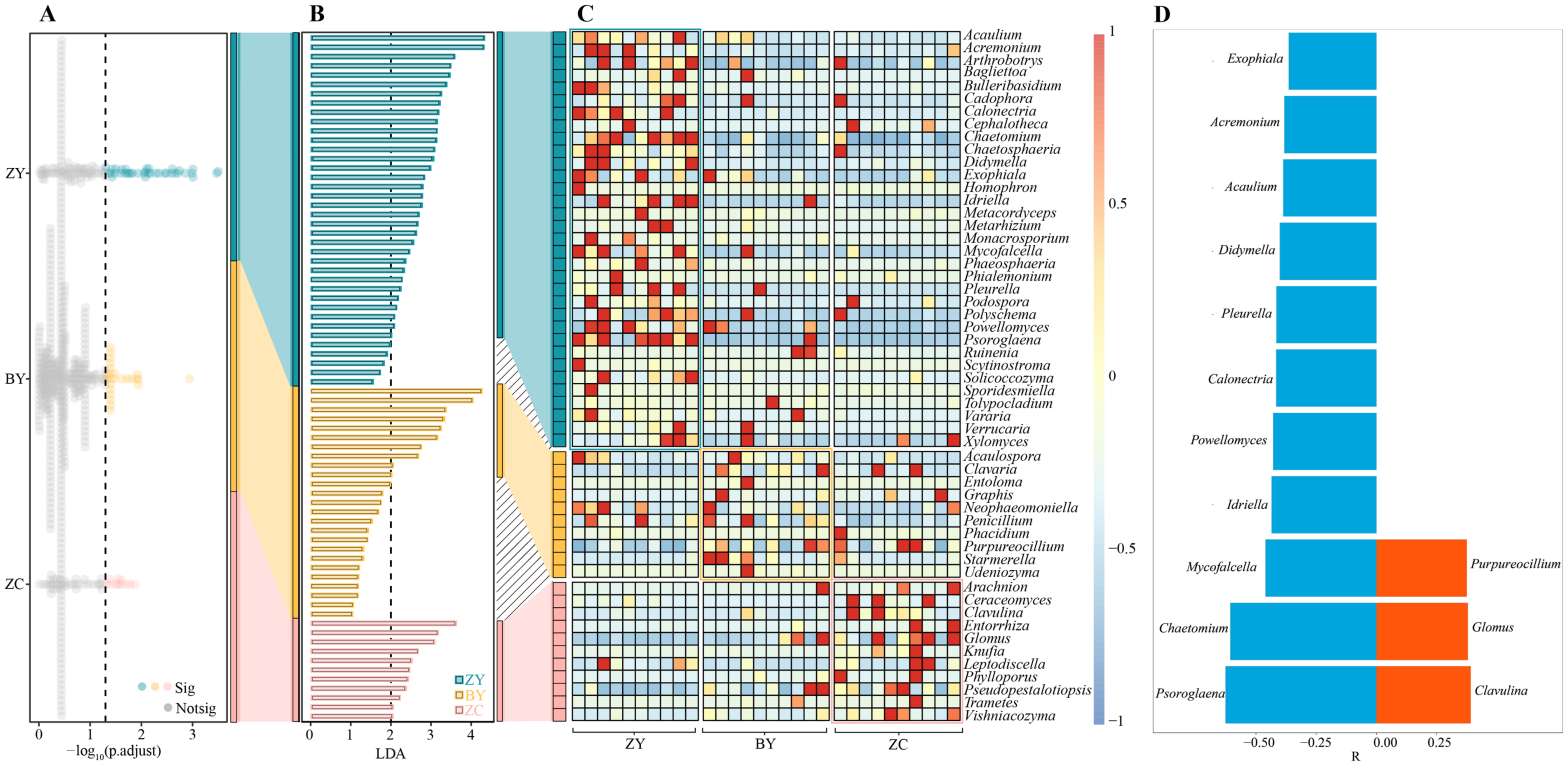
Kruskal Wallis test and linear discriminant analysis effect size (LEfSe) to screen for fungi with significant differences in abundance in tea plant rhizosphere soil in authentic rocky zone (ZY), semi-rock zone (BY) and continent zone (ZC) tea plantations. **(A)** Kruskal-Wallis test for average fungal abundance; **(B)** Linear discriminant analysis effect size (LEfSe) analysis of fungi that significantly distinguished ZY, BY, and ZC; **(C)** Heat map analysis of changes in differential fungal abundance; **(D)** Trend analysis of changes in differential fungal abundance (a negative value of relative coefficient (R) indicates a significant decreasing trend in fungal abundance from the authentic zone to the semi-rock zone to the continent zone, whereas a positive value indicates a significant increasing trend).

In addition, the present study also screened the fungi that differed significantly in tea plant rhizosphere soil in different rocky zones. The Kruskal-Wallis test (Figure 6A) revealed that 74 fungal genera were significantly different from each other in different rocky zones, and the LDA method of LEfSe (*p* < 0.05 and LDA > 2) revealed that 54 of the 74 genera could be significantly differentiated from ZY, BY, and ZC (Figure 6B), and significant differences existed in abundance between different rocky zones (Figure 6C). Further trend analysis of the 54 fungal genera revealed (Figure 6D) that 14 fungal genera showed regular changes from authentic rocky zone to semi-rock zone to continent zone, of which 3 fungal genera indicated a significant upward trend and 11 fungal genera indicated a significant downward trend. It is evident that the differences in rock zones still resulted in significant changes in fungal abundance in tea plant rhizosphere soil.

### 3.6 Screening of characteristic microorganisms in the rhizosphere soil of tea plant in different rocky zones

**Figure 7:**
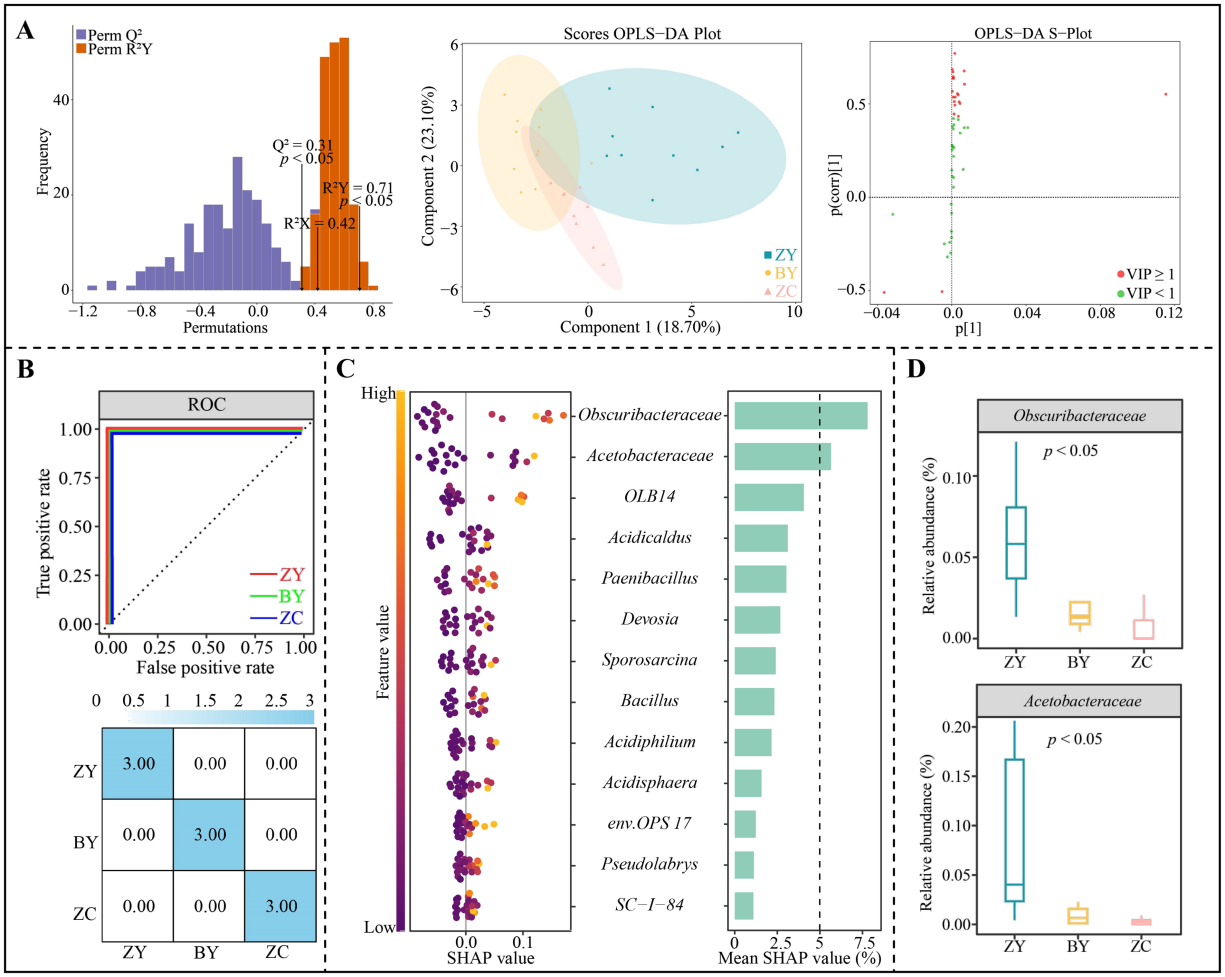
Orthogonal partial least squares discriminant analysis (OPLS-DA) model and random forest machine deep learning to screen and distinguish characteristic bacteria in tea plant rhizosphere soil from authentic rocky zone (ZY), semi-rock zone (BY) and continent zone (ZC) tea plantations. **(A)** Constructed OPLS-DA models for ZY, BY, and ZC using 50 significantly different bacterial genera to screen key differential bacteria; **(B)** Random forest deep machine learning simulation to analyse the accuracy of key differential bacteria in distinguishing ZY, BY and ZC; **(C)** SHAP analysis of random forest to obtain the characteristic bacteria in distinguishing ZY, BY and ZC; **(D)** Median analysis of characteristic bacterial abundance.

Based on the aforementioned analysis, in-depth study screened characteristic microorganisms of tea plant rhizosphere soil that could effectively distinguish different rocky zones. First, the OPLS-DA model of ZY, BY and ZC was constructed according to the abundance of 50 bacterial genera with significant differences. The results indicated (Figure 7A) that the fit (*R*^2^Y = 0.71) and predictability (Q^2^ = 0.31) of the model reached a significant level (*p* < 0.05), and that the model could effectively differentiate between ZY, BY and ZC. The 13 genera of key bacteria (VIP > 1) that could significantly differentiate ZY, BY and ZC were derived from the constructed OPLS-DA model. Further machine learning simulations were performed using random forests, and the results indicated (Figure 7B) that the overall accuracy and single classification accuracy of the machine learning simulations reached 100%. It can be seen that the 13 genera of key bacteria could effectively distinguish ZY, BY and ZC. Therefore, further SHAP value analysis revealed (Figure 7C) that for the SHAP values of the 13 genera of key bacteria in distinguishing ZY, BY and ZC, and the characteristic bacteria with greater than 5% or more were only two genera, namely *Obscuribacteraceae* and *Acetobacteraceae*. The abundance analysis of the characteristic bacteria showed (Figure 7D) that a significant decreasing trend existed in the abundance of the 2 bacterial genera from the authentic rocky zone to the semi-rock zone to the continent zone.

**Figure 8:**
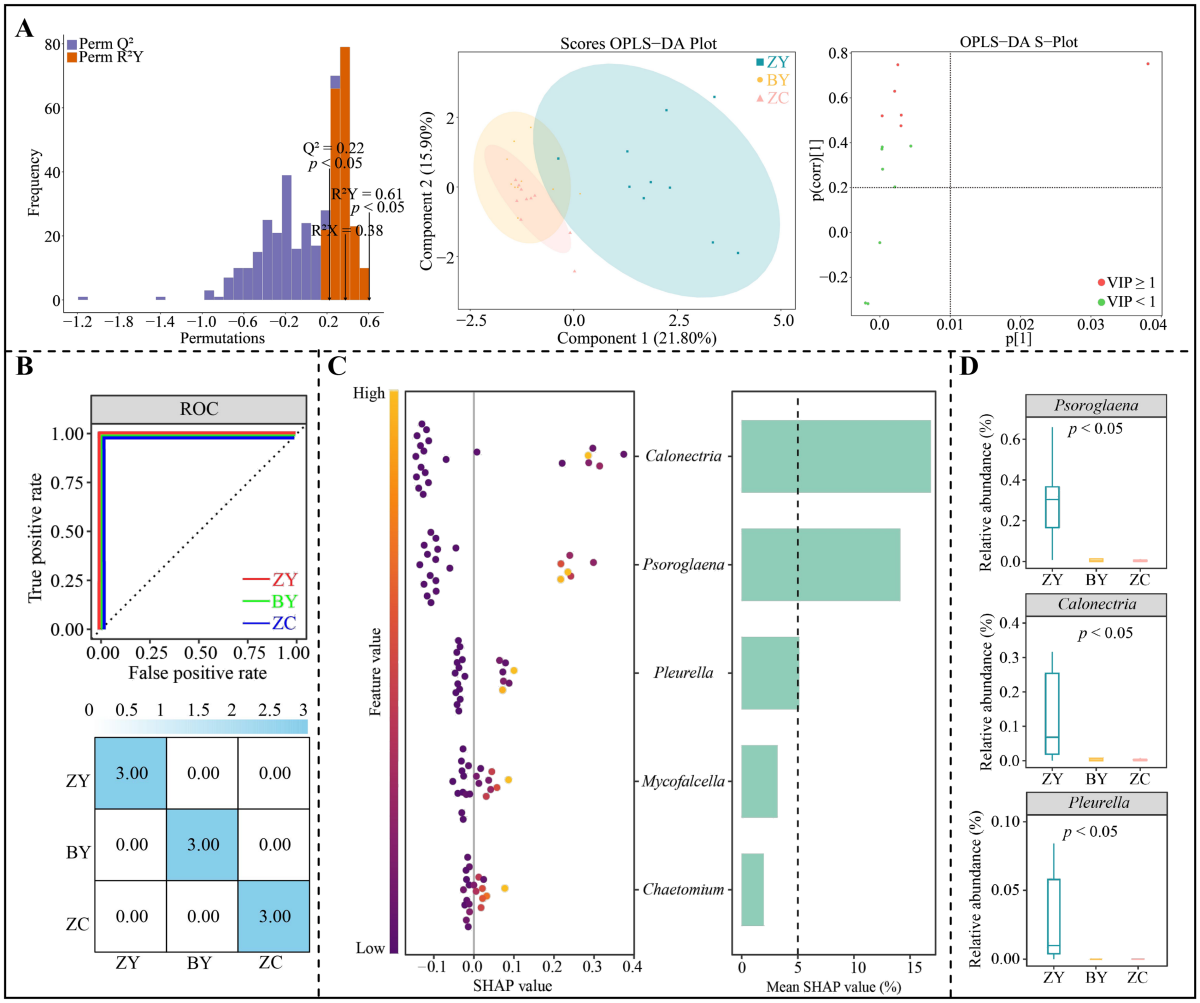
Orthogonal partial least squares discriminant analysis (OPLS-DA) model and random forest machine deep learning to screen and distinguish characteristic fungi in tea plant rhizosphere soil from authentic rocky zone (ZY), semi-rock zone (BY) and continent zone (ZC) tea plantations. **(A)** Constructed OPLS-DA models for ZY, BY, and ZC using 14 significantly different fungal genera to screen key differential fungi; **(B)** Random forest deep machine learning simulation to analyse the accuracy of key differential fungi in distinguishing ZY, BY and ZC; **(C)** SHAP analysis of random forest to obtain the characteristic fungi in distinguishing ZY, BY and ZC; **(D)** Median analysis of characteristic fungal abundance.

In addition, OPLS-DA models were constructed to distinguish ZY, BY and ZC based on the abundance of 14 fungal genera that differed significantly, and the results indicated (Figure 8A) that the OPLS-DA model reached a significant level of goodness of fit (*R*^2^Y = 0.61) and predictability (Q^2^ = 0.22) (*p* < 0.05), which could effective distinguish ZY, BY and ZC. A total of five key fungal genera (VIP > 1) that could significantly differentiate ZY, BY and ZC were derived from the constructed OPLS-DA model. Further machine learning simulation using random forest was found (Figure 8B), and the overall accuracy and single classification accuracy of the machine learning simulation all reached 100%. It is evident that the 5 key fungal genera could effectively distinguish ZY, BY and ZC. Further SHAP value analysis of these key fungal genera indicated (Figure 8C) that for the SHAP values of the 5 key fungal genera in distinguishing ZY, BY and ZC, the characteristic fungi that were greater than 5% or more were only 3 genera, which were *Psoroglaena*, *Calonectria* and *Pleurella*. The abundance analysis of the characteristic fungi indicated (Figure 8D) that a significant decreasing trend existed in the abundance of all three genera from the authentic rocky zone to the semi-rock zone to the continent zone.

### 3.7 Functional analysis of characteristic microorganisms of the rhizosphere soil of tea plant in different rocky zones

**Figure 9:**
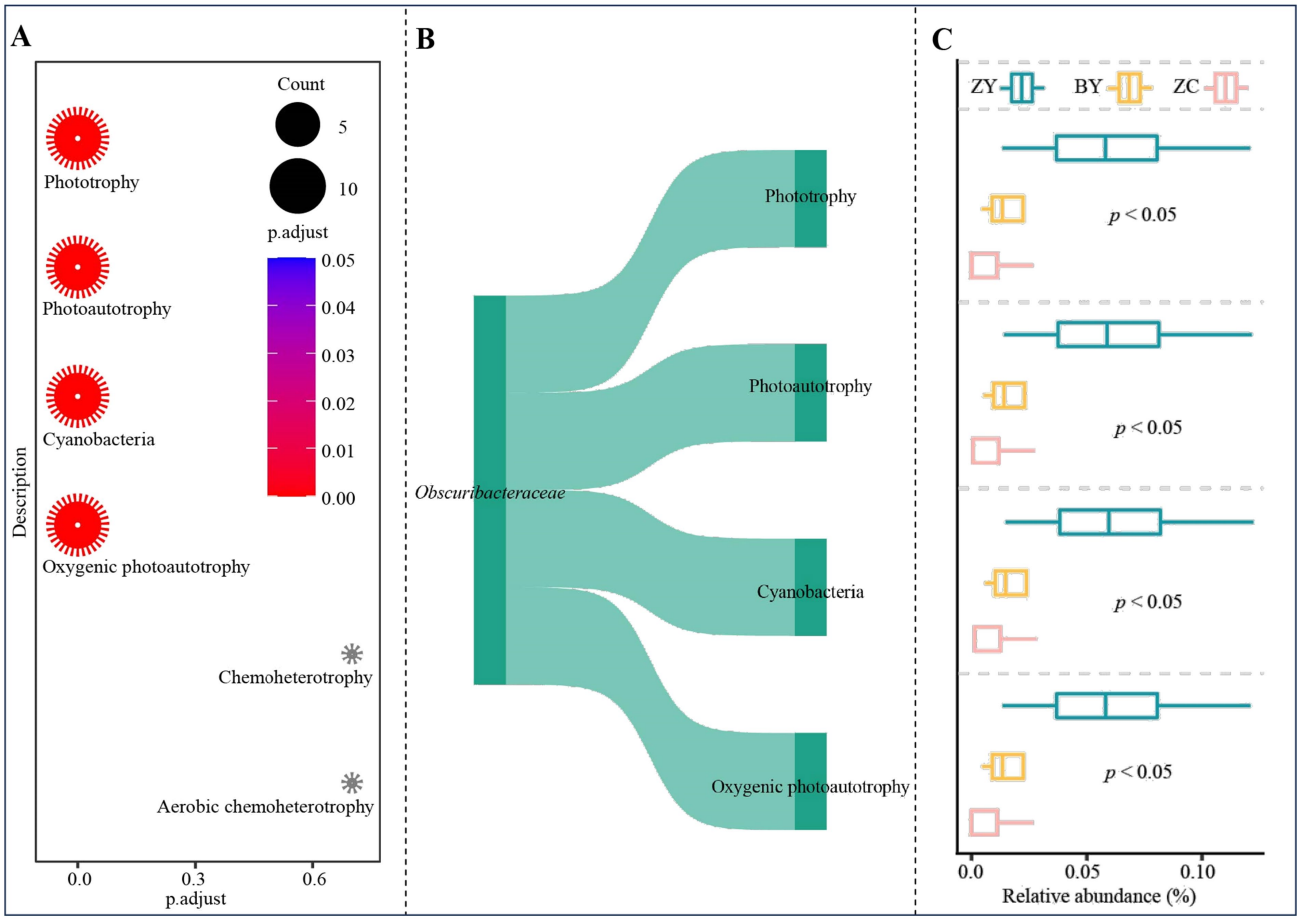
Functional prediction and enrichment analysis to obtain functions and their intensity differences of characteristic bacteria in tea plant rhizosphere soil in authentic rocky zone (ZY), semi-rock zone (BY) and continent zone (ZC) tea plantations. **(A)** Prediction and enrichment analysis of characteristic bacterial function using FUNGuild software; **(B)** OTUs of function significantly enriched by characteristic bacteria and their annotated bacterial analysis; **(C)** Analysis of functional intensity based on the abundance of OTUs significantly enriched in that function.

Based on the 2 genera of characteristic bacteria obtained from 58 OTUs, functional enrichment was carried out according to the functions annotated for each OTUs. The results indicated (Figure 9A) that there were 4 functions significantly enriched for characteristic bacteria, namely phototrophy, photoautotrophy, cyanobacteria, and oxygenic photoautotrophy. Further analysis (Figure 9B) revealed that the four functions significantly enriched were all from *Obscuribacteraceae*. The functional intensity analysis (Figure 9C) indicated that for the intensity of the four functions of the characteristic bacteria, ZY was significantly higher than BY and ZC, and BY was higher than ZC. It is evident that changes in the abundance of *Obscuribacteraceae* of soil characteristic bacteria in different rocky zones altered the functional intensity of soil bacteria.

**Figure 10:**
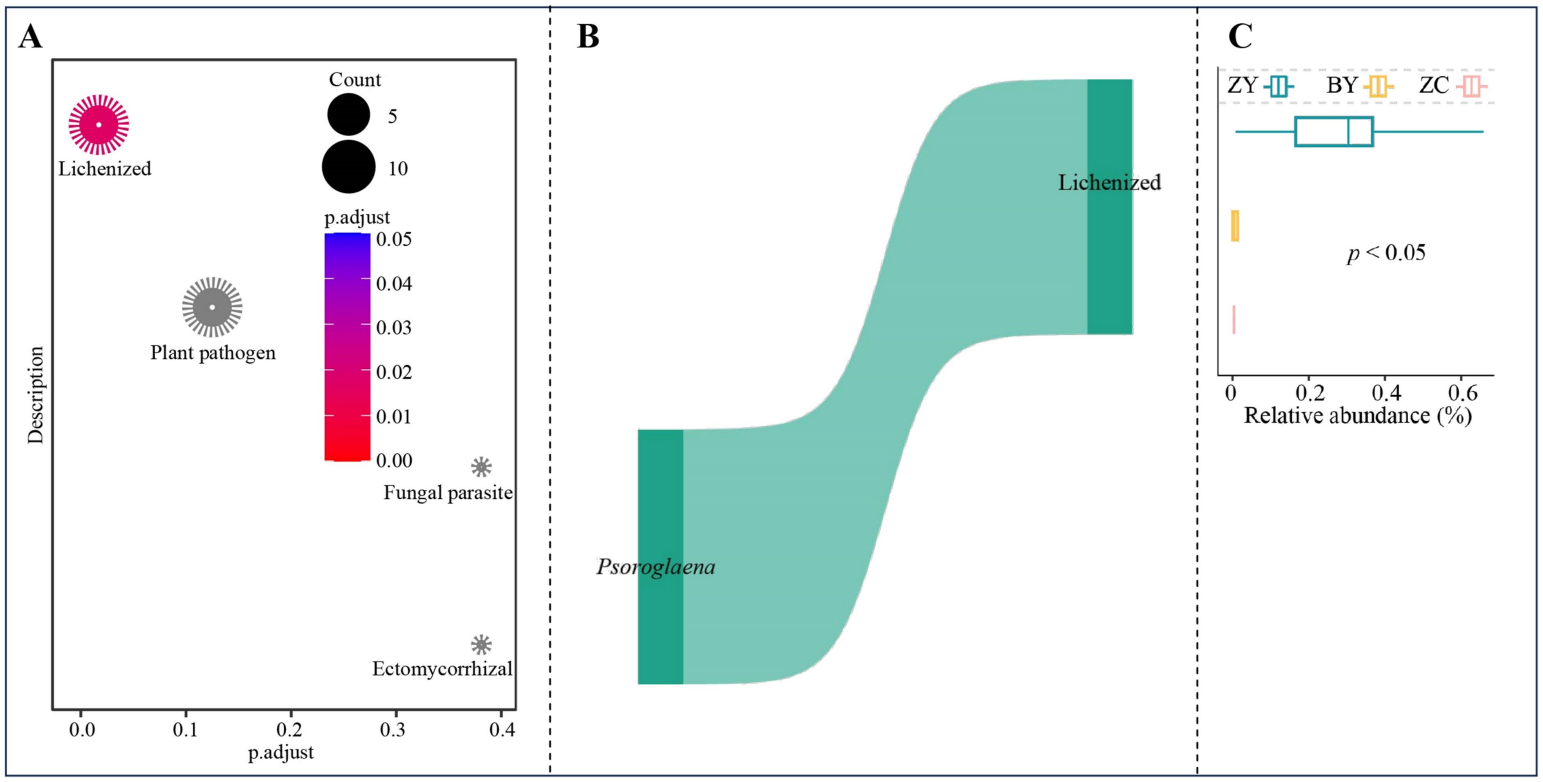
Functional prediction and enrichment analysis to obtain functions and their intensity differences of characteristic fungi in tea plant rhizosphere soil in authentic rocky zone (ZY), semi-rock zone (BY) and continent zone (ZC) tea plantations. **(A)** Prediction and enrichment analysis of characteristic fungi function using FUNGuild software; **(B)** OTUs of function significantly enriched by characteristic fungi and their annotated fungal analysis; **(C)** Analysis of functional intensity based on the abundance of OTUs significantly enriched in that function.

Based on the six OTUs corresponding to three characteristic fungal genera, functional enrichment analysis was performed using the function annotated for each OTUs, and the results indicated (Figure 10A) that the only function significantly enriched in characteristic fungi was lichenized. Further analysis revealed (Figure 10B) that the OTUs significantly enriched in lichenized function were all from Psoroglaena. Functional intensity analysis indicated (Figure 10C) that for the functional intensity of lichenized characteristic fungi, ZY was significantly greater than BY and ZC, while BY was greater than ZC. These findings indicate that changes in the abundance of *Psoroglaena* of soil characteristic fungi across different rocky zones altered the functional intensity of tea plant rhizosphere soil fungi.

### 3.8 Interaction analysis of different indices

Based on the above analysis, correlation network and PLS-SEM structural equations were further employed to explore relationships among the characteristic microorganisms, functional intensities and soil physicochemical indices and tea quality. Correlation network analysis indicated (Supplementary Figure S2A) that *Obscuribacteraceae* showed significant and positive correlation with the intensities of the four functions, including phototrophy, photoautotrophy, cyanobacteria, and oxygenic photoautotrophy. These four functions were significantly positively correlated with soil available nitrogen, available phosphorus, organic matter content and total phosphorus contents, and insignificant correlation with available potassium content. Secondly, soil available nitrogen, available potassium and available phosphorus contents were significantly and positively correlated with tea quality indices, while organic matter and total phosphorus contents displayed significant and negative correlation. Additionally, further analysis revealed (Supplementary Figure S2B) that *Psoroglaena,* the characteristic fungus, had significant and positive correlation with lichenized function, while lichenized existed significant and positive correlation with soil available nitrogen and available phosphorus content, significantly and negatively correlated with organic matter content and total phosphorus content, and not significantly correlated with available potassium content. Using PLS-SEM to construct structural equations for different indicators revealed (Supplementary Figure S2C) that the potential functional intensity of the characteristic bacteria *Obscuribacteraceae* and the characteristic fungi *Psoroglaena* both showed positive correlations with the physicochemical indicators of tea plant rhizosphere soil and tea leaf quality. This indicates that changes in the abundance of characteristic microorganisms across different rock zones may alter soil microbial functions, affecting nutrient transformation in the soil and consequently influencing tea leaf quality.

## 4 Discussion

The present study confirmed that there was indeed a significant difference in the quality indices of tea plant leaves in different rocky zones (ZY > BY>ZC), this result obtained was consistent with the study of Ali Abaker Omer et al.’s (2025) study on the major tea zones of China, which concluded that geography is an important factor influencing the chemical composition of tea leaves, and that ZY tea plantations have the best quality tea leaves. It is worth noting that although the available nutrient content of ZY soils was higher, their total phosphorus and organic matter contents were, on the contrary, lower than those of ZC, which was similar to the discovery of Tang et al. (2022), indicating that it was difficult to fully explain the differences of tea quality by soil nutrient content alone, implying that microbial factors might play a key role in quality formation. Microorganisms play a crucial role in soil nutrient cycling processes and maintaining soil health (Jibola-Shittu et al., 2024). For example, Lei et al. (2024) found that tea quality exhibited significant changes following different fertilizer treatments, and the key reason was the significant accumulation of microorganisms such as *Trichoderma* and *Fusarium oxysporum* in the rhizosphere soil, which enhanced the availability of nutrients in the soil and accelerated their uptake and accumulation by tea plants. Huang et al. (2023) observed that organic tea plantations exhibited significantly increased abundances of *Rhombodiales* and *Nitrospirae* in the rhizosphere soil, leading to stronger soil nutrient cycling capacity and higher tea leaf quality.

This study analysis from the microbial perspective revealed that the ZY microbial community was most affected by stochastic processes, suggesting that its community construction was more dependent on stochastic events such as biological dispersal. This instability may have created more ecological niche opportunities and promoted the colonization of functionally specialized microorganisms, such as *Obscuribacteraceae* found in this study. *Obscuribacteraceae* have been reported to be positively correlated with tea quality in rhizosphere studies of tea plants, and may influence the growth of tea plants through their specific metabolic functions (Zhang et al., 2025). In contrast, the ZC community indicated stronger deterministic selection, reflecting its stronger environmental filtering. This difference provided a new ecological perspective to explain the differences in tea quality among different rocky zones. Secondly, this study found that the ZC microbial network had the highest complexity but lower functional intensity. This result illustrated that the microbial diversity was enhanced and the functional types was increased, but on the contrary, the intensity of special functions was reduced (Hernandez et al., 2021). In the ZY zone, the abundance of key functional microorganisms was higher, although the average degree of network nodes was lower, a community structure dominated by “elite microorganisms,” which was similarly reported in the global soil microbiology study by Delgado-Baquerizo et al. (2018).

Secondly, *Obscuribacteraceae* and *Psoroglaena* of characteristic microorganisms obtained through machine learning screening in this study, have also been identified as potential biomarkers for high quality tea in tea plant microbiome studies (Costa et al., 2022; Seaward and Aptroot, 2006). *Obscuribacteraceae* are known to have carbon sequestration properties (Mamlouk and Gullo, 2013), and *Psoroglaena* has the property of promoting rock weathering and accelerating the release of mineral elements (McCarthy and Kantvilas, 2013), which may have a direct impact on tea quality by promoting nutrient uptake by the tea plant. This finding provided evidence for understanding the microbial mechanisms of “rocky flavor” formation.

In addition, functional analysis of the characteristic microorganisms indicated that the ZY characteristic bacteria were significantly enriched in four photosynthesis-related pathways, including phototrophy, and these functions were exclusively from the *Obscuribacteraceae*. This bacterium is known to be photosynthesizing and to secrete organic acids, such as acetic acid. Patel et al. (2024), in a study of microbial function in rocky habitats also found a significant increase in the abundance of bacteria in this genus. In the typical low-light environment of ZY, these photosynthetic microorganisms might play the role of “primary producers” and provided an additional energy for the rhizosphere micro-ecosystem. For the characteristic fungus *Psoroglaena*, its enriched lichenized function suggested that it might have lichen symbiosis. In the typical rock-soil interface environment of ZY, this group of fungi may be involved in the rock weathering process and promote the release of mineral elements. Halda et al. (2020) also found that *Psoroglaena* played an important role in the weathering of rocks, and this result also explained why ZY tea had a higher mineral content (Zhang et al., 2024). These findings provided new microbiological evidence to explain the material basis of “rock flavor.”

**Figure 11:**
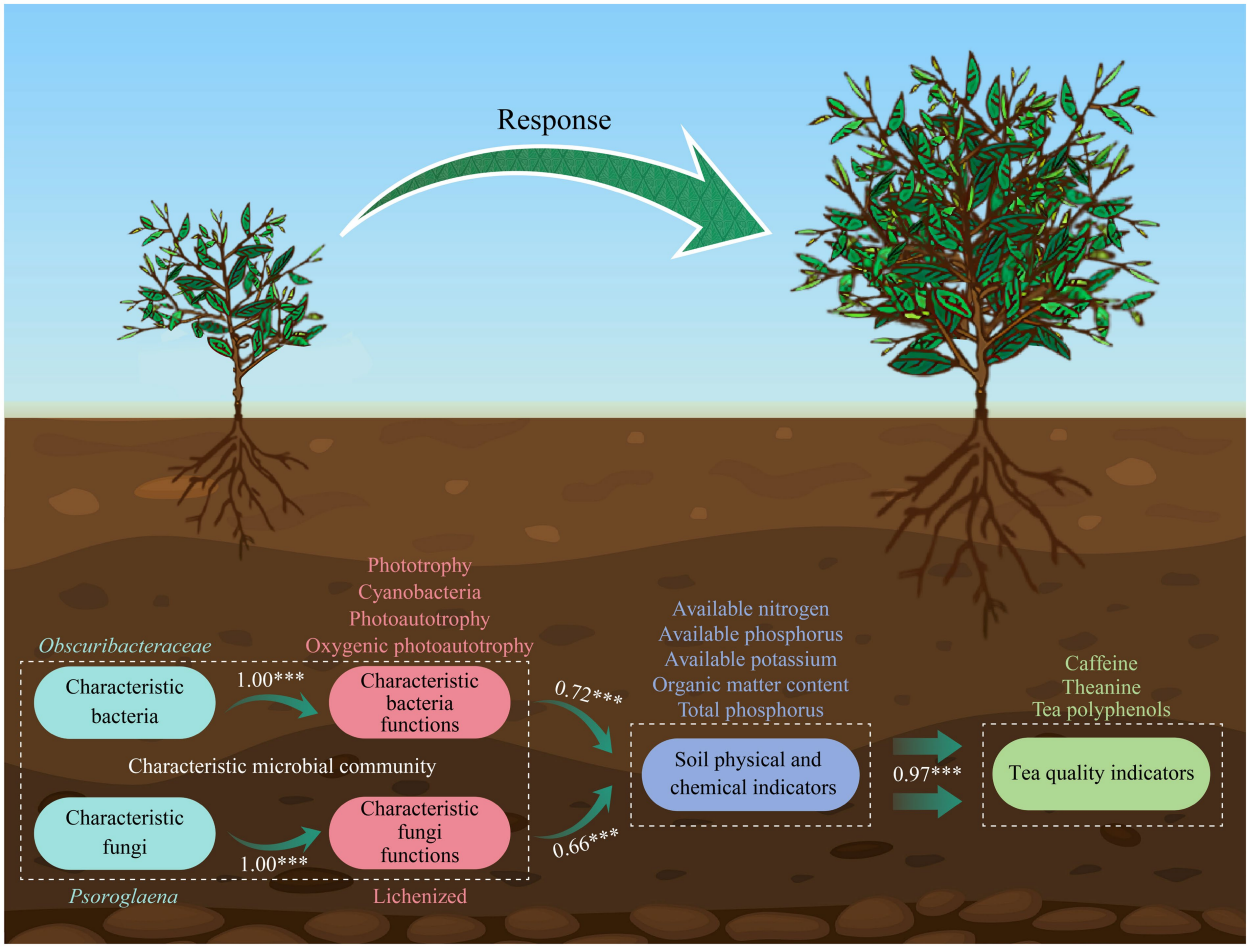
Model of characteristic microorganisms regulating soil nutrient transformation to improve tea quality.

In conclusion, this study systematically compared the differences in leaf quality indices, rhizosphere soil physicochemical properties and microbial community property of ZY, BY and ZC tea plants, revealing the integrated effects of rock zone types on the micro-ecosystems of tea plant growth. This study found (Figure 11) that the rhizosphere microbial community of tea plant in ZY was more affected by stochastic processes, with higher functional intensity but simpler network structure; the abundance of *Obscuribacteraceae* and *Psoroglaena* of characteristic microorganisms was positively correlated with tea quality; and characteristic microorganisms might be involved in the formation of the “rock flavor” through the special functions such as photosynthesis and rock weathering. These findings provide microbial ecological explanations for the formation of regional characteristics of Wuyi rock tea, and provide new insights into the formation mechanism of such differences from the perspective of microbial ecology, which lays a theoretical foundation for subsequent research and the development of Wuyi rock tea industry. However, beyond soil variations, different rock zones also exhibit distinct natural environmental conditions. These differences can influence tea quality to a certain extent, necessitating further exploration in subsequent studies. Additionally, the microorganisms identified in this study as relevant to tea quality require further isolation, purification, and functional validation in future research. This will provide valuable insights for developing microbial fertilizers tailored for tea plantations.

## Data Availability

The datasets presented in this study can be found in online repositories. The names of the repository/repositories and accession number(s) can be found in the article/Supplementary material.
