# Supplementary material for: Differences in abundance and functional intensity of characteristic microorganisms of tea plant rhizosphere soils contribute to the differentiation of tea quality in different rocky zones
Source: Front Microbiol. 2025 Nov 14;16:1704146. doi: 10.3389/fmicb.2025.1704146 (PMC12660103; doi:10.3389/fmicb.2025.1704146)
Supplement: Supplementary file 1 [file Data_Sheet_1.PDF]

Supplementary data

1. Figure S1. OTUs analysis of rhizosphere soil bacteria and fungi of tea plant in different rocky zones. Note: ZY: Authentic rocky zone; BY: Semi-rock zone; ZC: Continent zone; 1~10 represent 10 different tea plantations in this rock zone. (A) Rarefaction curve analysis of bacterial OTUs; (B) Shannon-wiener curve analysis of bacterial diversity; (C) Rank-abundance curve graph of changes in bacterial abundance; (D) Species accumulation curves of bacteria; (E) Rarefaction curve analysis of fungal OTUs; (F) Shannon-wiener curve analysis of fungal diversity; (G) Rank-abundance curve graph of changes in fungal abundance; (H) Species accumulation curve graph of fungi;
2. Figure S2. Interaction analysis of characteristic microorganisms and different indices. Note: (A) Correlation network analysis of characteristic bacteria, functional intensities, soil physicochemical indices and tea quality indices; (B) Correlation network analysis of characteristic fungi, functional intensities, soil physicochemical indices and tea quality indices; (C) Construction of PLS-SEM equations for characteristic microorganisms, functional intensities, soil physicochemical indices and tea quality indices;
3. Table S2. Distribution of high-quality sequences after sequencing of rhizosphere soil bacteria of tea plant in different rocky zones;
4. Table S3. Statistics of the number of bacterial OTUs in rhizosphere soils of tea plant in different rocky zones;
5. Table S4. Statistics of splicing results after sequencing of rhizosphere fungi of tea plant in different rocky zones;
6. Table S5. Distribution of high-quality sequences after sequencing of rhizosphere soil fungi of tea plant in different rocky zones;
7. Table S6. Statistics of the number of OTUs of fungi in rhizosphere soils of tea plant in different rocky zones;
8. Table S7. Analysis of the diversity indices of the bacterial community in rhizosphere soil of tea plant in different rocky zones;
9. Table S8. Analysis of the diversity indices of the fungal community in rhizosphere soil of tea plant in different rocky zones.

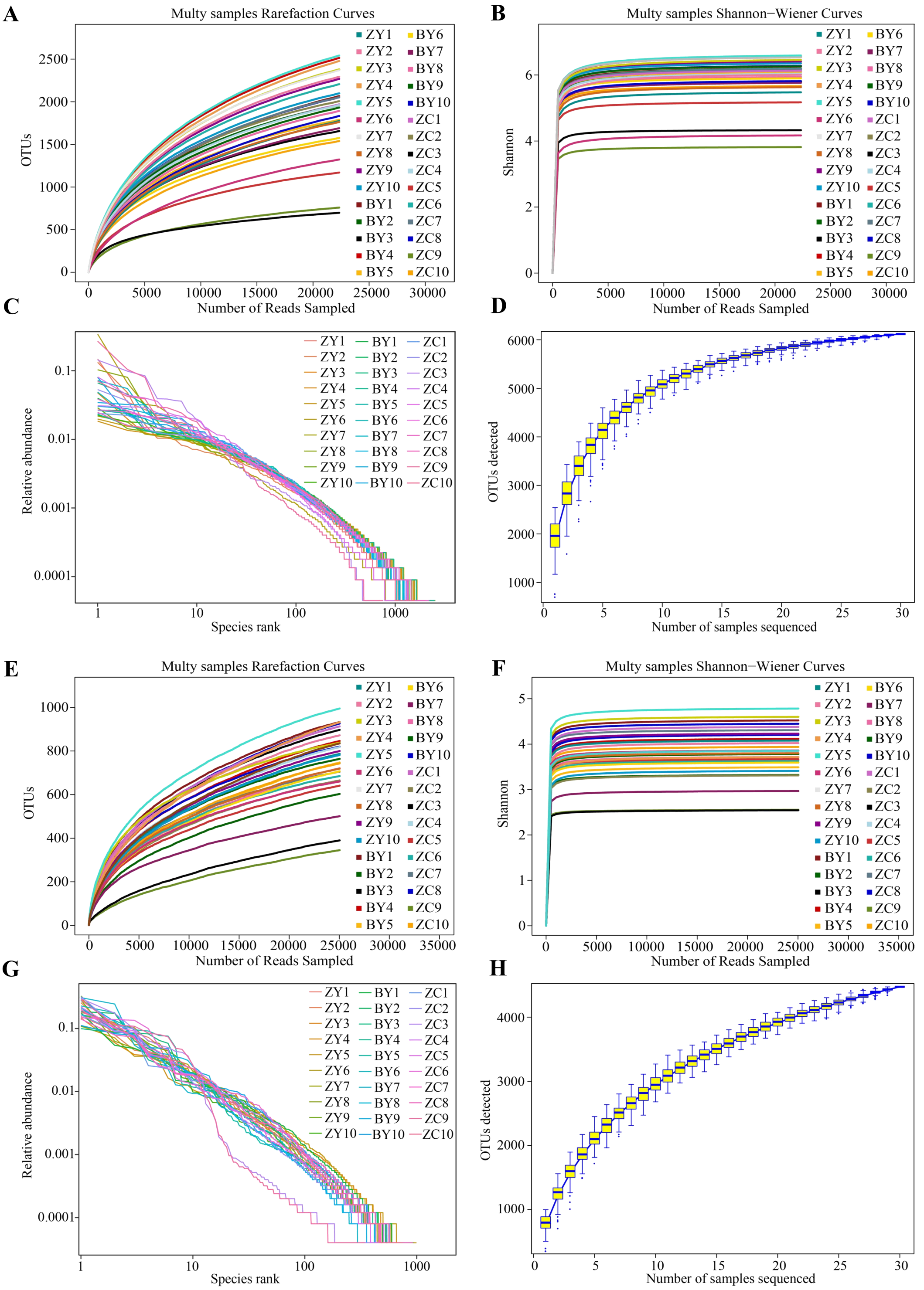

Figure S1. OTUs analysis of rhizosphere soil bacteria and fungi of tea plant in different rocky zones. Note: ZY: Authentic rocky zone; BY: Semi-rock zone; ZC: Continent zone; 1~10 represent 10 different tea plantations in this rock zone. (A) Rarefaction curve analysis of bacterial OTUs; (B) Shannon-wiener curve analysis of bacterial diversity; (C) Rank-abundance curve graph of changes in bacterial abundance; (D) Species accumulation curves of bacteria; (E) Rarefaction curve analysis of fungal OTUs; (F) Shannon-wiener curve analysis of fungal diversity; (G) Rank-abundance curve graph of changes in fungal abundance; (H) Species accumulation curve graph of fungi.

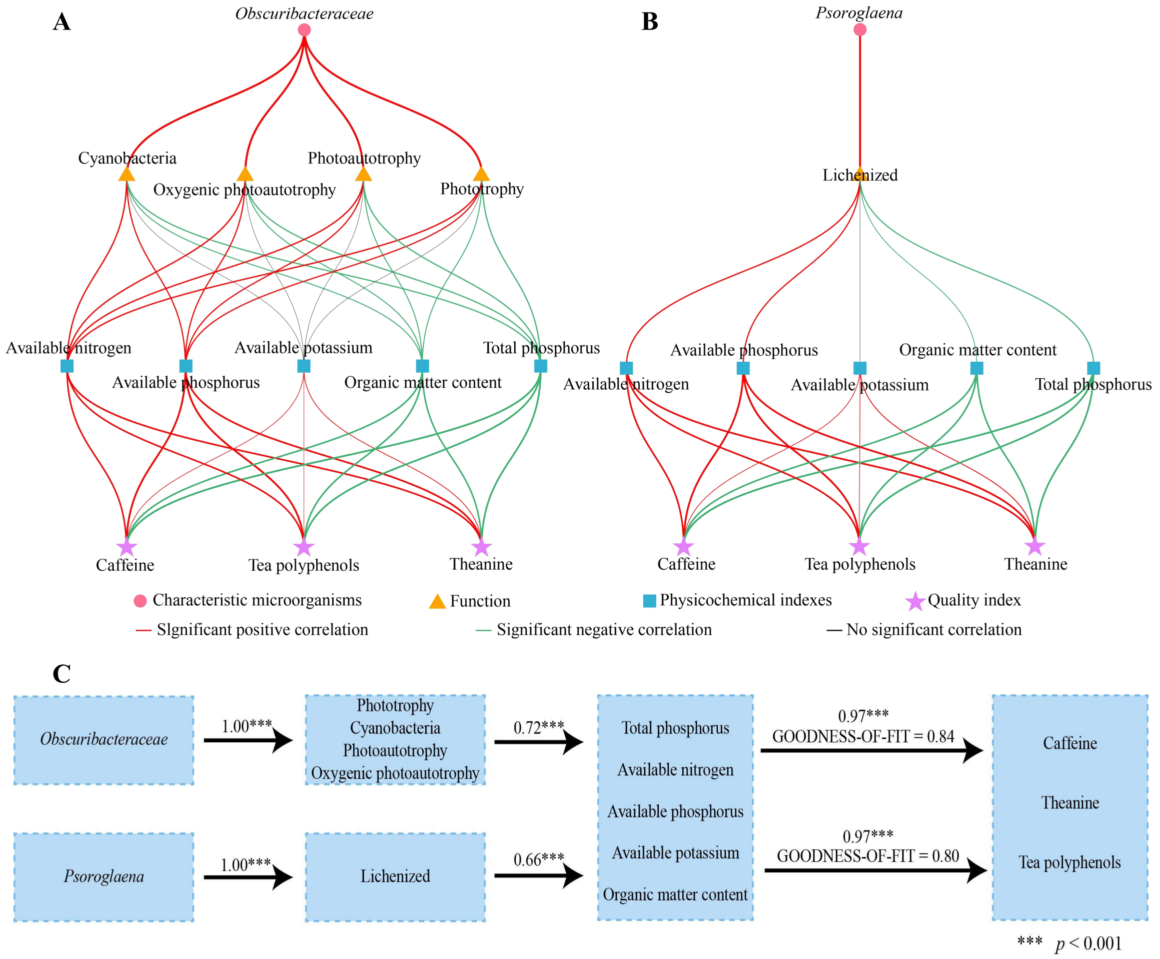

Figure S2. Interaction analysis of characteristic microorganisms and different indices. Note: (A) Correlation network analysis of characteristic bacteria, functional intensities, soil physicochemical indices and tea quality indices; (B) Correlation network analysis of characteristic fungi, functional intensities, soil physicochemical indices and tea quality indices; (C) Construction of PLS-SEM equations for characteristic microorganisms, functional intensities, soil physicochemical indices and tea quality indices.

Table S1. Statistics of splicing results after sequencing of rhizosphere soil bacteria of tea plant in different rocky zones

Sample ID	Raw tags (Mb)	Clean tags (Mb)	Clean/Raw tags ratio	Sample ID	Raw tags (Mb)	Clean tags (Mb)	Clean/Raw tags ratio	Sample ID	Raw tags (Mb)	Clean tags (Mb)	Clean/Raw tags ratio
**ZY1**	80876	77668	96.03%	**BY1**	42444	39776	93.71%	**ZC1**	45914	44432	96.77%
**ZY2**	35142	32512	92.52%	**BY2**	58041	56267	96.94%	**ZC2**	88199	81319	92.20%
**ZY3**	31380	30807	98.17%	**BY3**	73584	67697	92.00%	**ZC3**	30962	30026	96.98%
**ZY4**	43126	40015	92.79%	**BY4**	33377	31778	95.21%	**ZC4**	50136	48054	95.85%
**ZY5**	82831	78611	94.91%	**BY5**	48103	46734	97.15%	**ZC5**	46222	42115	91.11%
**ZY6**	34897	32702	93.71%	**BY6**	46620	45574	97.76%	**ZC6**	92901	83392	89.76%
**ZY7**	69167	62595	90.50%	**BY7**	34277	31556	92.06%	**ZC7**	35813	34599	96.61%
**ZY8**	69403	66047	95.16%	**BY8**	48303	47169	97.65%	**ZC8**	41050	39269	95.66%
**ZY9**	43139	40371	93.58%	**BY9**	55035	53112	96.51%	**ZC9**	34038	33549	98.56%
**ZY10**	84605	80516	95.17%	**BY10**	40150	38762	96.54%	**ZC10**	43824	42730	97.50%
**Total raw tags**							1,563,559 Mb				
**Total rlean tags**							1,479,754 Mb				
**Total Clean/Raw tags ratio**							94.97%				

Note: ZY: authentic rock zone; BY: semi-rock zone; ZC: continent zone; 1~10 represent 10 different tea plantations in this rock zone.

Table S2. Distribution of high-quality sequences after sequencing of rhizosphere soil bacteria of tea plant in different rocky zones

Length (bp) distribute	Sequences reads
0-200	0
200-260	911
260-320	731
320-360	570
360-380	351
380-400	31,420
400-420	1,108,915
420-440	335,578
440-460	544
460-480	465
480-500	129
500-520	89
520-540	51
540-560	0
560-600	0

Table S3. Statistics of the number of bacterial OTUs in rhizosphere soils of tea plant in different rocky zones

Sample ID	Final tags	OTUs	Sample ID	Final tags	OTUs	Sample ID	Final tags	OTUs
**ZY1**	22,331	2,293	**BY1**	22331	2,067	**ZC1**	22331	1,970
**ZY2**	22,331	2,380	**BY2**	22331	1,959	**ZC2**	22331	2,007
**ZY3**	22,331	2,478	**BY3**	22331	1,656	**ZC3**	22331	698
**ZY4**	22,331	2,542	**BY4**	22331	2,514	**ZC4**	22331	1,961
**ZY5**	22,331	1,323	**BY5**	22331	1,788	**ZC5**	22331	1,170
**ZY6**	22,331	2,369	**BY6**	22331	1,575	**ZC6**	22331	2,206
**ZY7**	22,331	1,762	**BY7**	22331	1,690	**ZC7**	22331	2,053
**ZY8**	22,331	2,268	**BY8**	22331	1,892	**ZC8**	22331	2,052
**ZY9**	22,331	2,099	**BY9**	22331	1,932	**ZC9**	22331	759
**ZY10**	22,331	2,293	**BY10**	22331	1,833	**ZC10**	22331	1,539

Note: ZY: authentic rock zone; BY: semi-rock zone; ZC: continent zone; 1~10 represent 10 different tea plantations in this rock zone.

Table S4. Statistics of splicing results after sequencing of rhizosphere fungi of tea plant in different rocky zones

Sample ID	Raw tags (Mb)	Clean tags (Mb)	Clean/Raw tags ratio	Sample ID	Raw tags (Mb)	Clean tags (Mb)	Clean/Raw tags ratio	Sample ID	Raw tags (Mb)	Clean tags (Mb)	Clean/Raw tags ratio
**ZY1**	80,851	78,562	97.17%	**BY1**	60,757	56,751	93.41%	**ZC1**	52,493	48,066	91.57%
**ZY2**	52,201	50,056	95.89%	**BY2**	87,396	85,535	97.87%	**ZC2**	86,919	85,490	98.36%
**ZY3**	87,833	85,047	96.83%	**BY3**	86,847	84,660	97.48%	**ZC3**	38,760	37,195	95.96%
**ZY4**	83,728	81,387	97.20%	**BY4**	57,274	55,539	96.97%	**ZC4**	55,913	50,361	90.07%
**ZY5**	83,288	80,381	96.51%	**BY5**	88,128	87,035	98.76%	**ZC5**	86,878	80,724	92.92%
**ZY6**	82,889	78,238	94.39%	**BY6**	88,601	84,998	95.93%	**ZC6**	87,402	85,369	97.67%
**ZY7**	81,993	78,223	95.40%	**BY7**	87,467	83,730	95.73%	**ZC7**	45,540	43,231	94.93%
**ZY8**	85,237	81,665	95.81%	**BY8**	88,718	68,676	77.41%	**ZC8**	53,729	52,330	97.40%
**ZY9**	80,747	77,928	96.51%	**BY9**	75,261	71,784	95.38%	**ZC9**	49,224	48,994	99.53%
**ZY10**	83,279	81,095	97.38%	**BY10**	27,237	25,456	93.46%	**ZC10**	70,821	69,636	98.33%
**Total raw tags**							2,177,411 Mb				
**Total rlean tags**							2,078,142 Mb				
**Total Clean/Raw tags ratio**							95.41%				

Note: ZY: authentic rock zone; BY: semi-rock zone; ZC: continent zone; 1~10 represent 10 different tea plantations in this rock zone.

Table S5. Distribution of high-quality sequences after sequencing of rhizosphere soil fungi of tea plant in different rocky zones

Length (bp) distribute	Sequences reads
0-200	411,124
200-260	1,133,473
260-320	463,670
320-360	29,600
360-380	16,936
380-400	3,559
400-420	7,250
420-440	1,308
440-460	2,640
460-480	1,611
480-500	5,261
500-520	1,280
520-540	430
540-560	0
560-600	0

Table S6. Statistics of the number of OTUs of fungi in rhizosphere soils of tea plant in different rocky zones

Sample ID	Final tags	OTUs	Sample ID	Final tags	OTUs	Sample ID	Final tags	OTUs
**ZY1**	25,036	787	**BY1**	25,036	933	**ZC1**	25,036	913
**ZY2**	25,036	872	**BY2**	25,036	764	**ZC2**	25,036	665
**ZY3**	25,036	838	**BY3**	25,036	897	**ZC3**	25,036	390
**ZY4**	25,036	932	**BY4**	25,036	846	**ZC4**	25,036	818
**ZY5**	25,036	995	**BY5**	25,036	747	**ZC5**	25,036	641
**ZY6**	25,036	660	**BY6**	25,036	707	**ZC6**	25,036	685
**ZY7**	25,036	835	**BY7**	25,036	501	**ZC7**	25,036	705
**ZY8**	25,036	719	**BY8**	25,036	721	**ZC8**	25,036	926
**ZY9**	25,036	800	**BY9**	25,036	603	**ZC9**	25,036	345
**ZY10**	25,036	782	**BY10**	25,036	820	**ZC10**	25,036	743

Note: ZY: authentic rock zone; BY: semi-rock zone; ZC: continent zone; 1~10 represent 10 different tea plantations in this rock zone.

Table S7. Analysis of the diversity indices of the bacterial community in rhizosphere soil of tea plant in different rocky zones

Sample ID	Chao1	PD whole tree	Shannon	Simpson	Sample ID	Chao1	PD whole tree	Shannon	Simpson	Sample ID	Chao1	PD whole tree	Shannon	Simpson
**ZY1**	2,740.32	113.55	7.90	0.97	**BY1**	2,989.21	124.78	8.95	0.99	**ZC1**	2,653.11	132.11	8.84	0.99
**ZY2**	3,076.09	137.37	8.66	0.98	**BY2**	2,570.86	121.51	9.03	1.00	**ZC2**	2,656.00	121.44	8.88	0.99
**ZY3**	3,236.21	139.47	9.31	0.99	**BY3**	2,325.90	129.18	8.36	0.99	**ZC3**	1,119.67	73.86	6.24	0.95
**ZY4**	3,473.80	145.51	9.47	1.00	**BY4**	3,411.48	148.01	9.44	1.00	**ZC4**	2,684.67	129.28	8.71	0.99
**ZY5**	3,423.40	151.22	9.51	1.00	**BY5**	2,389.07	121.02	8.79	0.99	**ZC5**	1,591.98	80.32	7.46	0.98
**ZY6**	1,958.29	90.94	6.01	0.87	**BY6**	2,251.25	99.68	8.51	0.99	**ZC6**	3,027.36	135.84	8.93	0.99
**ZY7**	3,306.02	138.41	9.43	1.00	**BY7**	2,377.19	118.09	8.31	0.99	**ZC7**	2,798.67	126.36	8.90	0.99
**ZY8**	2,509.23	113.9	8.12	0.98	**BY8**	2,628.13	116.64	8.57	0.99	**ZC8**	2,862.57	133.88	9.05	1.00
**ZY9**	3,116.31	136.03	9.24	1.00	**BY9**	2,705.74	117.25	8.79	0.99	**ZC9**	1,196.53	79.36	5.50	0.9
**ZY10**	2,851.26	128.34	9.16	1.00	**BY10**	2,732.03	116.02	8.39	0.99	**ZC10**	2,090.35	108.05	8.17	0.99

Note: ZY: authentic rock zone; BY: semi-rock zone; ZC: continent zone; 1~10 represent 10 different tea plantations in this rock zone.

Table S8. Analysis of the diversity indices of the fungal community in rhizosphere soil of tea plant in different rocky zones

Sample ID	Chao1	PD whole tree	Shannon	Simpson	Sample ID	Chao1	PD whole tree	Shannon	Simpson	Sample ID	Chao1	PD whole tree	Shannon	Simpson
**ZY1**	1,298.89	167.37	5.88	0.95	**BY1**	1,314.07	172.81	6.52	0.96	**ZC1**	1,304.78	174.10	6.32	0.95
**ZY2**	1,216.83	168.25	5.80	0.92	**BY2**	1,027.02	163.48	5.45	0.92	**ZC2**	919.40	126.88	4.78	0.87
**ZY3**	1,113.03	190.75	6.64	0.96	**BY3**	1,327.11	178.04	6.09	0.96	**ZC3**	759.34	93.97	3.67	0.85
**ZY4**	1,462.78	181.79	5.36	0.89	**BY4**	1,269.61	169.26	5.94	0.94	**ZC4**	1,184.96	162.00	5.88	0.94
**ZY5**	1,353.32	194.82	6.90	0.97	**BY5**	1,273.19	147.24	5.03	0.92	**ZC5**	1,013.57	135.04	5.29	0.92
**ZY6**	880.65	138.25	5.57	0.94	**BY6**	1,131.95	138.95	5.18	0.9	**ZC6**	937.83	149.35	5.24	0.92
**ZY7**	1,281.58	168.88	5.56	0.90	**BY7**	940.90	106.34	4.28	0.84	**ZC7**	971.78	142.86	6.21	0.96
**ZY8**	1,042.79	150.87	5.48	0.91	**BY8**	1,181.88	143.25	5.36	0.93	**ZC8**	1,389.79	179.57	6.06	0.94
**ZY9**	1,116.30	154.89	6.10	0.96	**BY9**	915.57	136.47	4.79	0.89	**ZC9**	699.58	82.23	3.68	0.87
**ZY10**	1,085.55	158.32	4.92	0.87	**BY10**	1,165.92	157.63	6.42	0.97	**ZC10**	1,053.78	159.28	5.68	0.94

Note: ZY: authentic rock zone; BY: semi-rock zone; ZC: continent zone; 1~10 represent 10 different tea plantations in this rock zone.
